# Citrinin Dietary Exposure Assessment Approach through Human Biomonitoring High-Resolution Mass Spectrometry-Based Data

**DOI:** 10.1021/acs.jafc.1c01776

**Published:** 2021-06-01

**Authors:** Alfonso Narváez, Luana Izzo, Yelko Rodríguez-Carrasco, Alberto Ritieni

**Affiliations:** † Department of Pharmacy, Faculty of Pharmacy, University of Naples “Federico II”, Via Domenico Montesano 49, Naples 80131, Italy; ‡ Laboratory of Food Chemistry and Toxicology, Faculty of Pharmacy, University of Valencia, Av. Vicent Andrés Estellés s/n, Burjassot, València 46100, Spain; § UNESCO Chair on Health Education and Sustainable Development, “Federico II” University, Naples 80131, Italy

**Keywords:** Orbitrap, biomarkers, exposure, citrinin, biomonitoring, urine

## Abstract

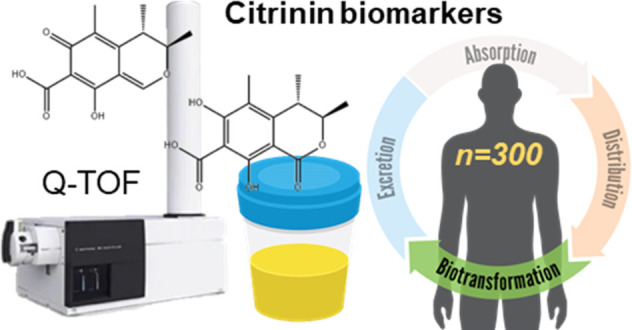

Citrinin (CIT) is a scarcely studied mycotoxin within foodstuffs, so the biomonitoring of this toxin and its metabolite dihydrocitrinone (DH-CIT) in biological samples represents the main alternative to estimate the exposure. Hence, this study aimed to evaluate the presence of CIT and DH-CIT in 300 urine samples from Italian individuals in order to assess the exposure. Quantification was performed through an ultrahigh-performance liquid chromatography high-resolution mass spectrometry (UHPLC-Q-Orbitrap HRMS)-based methodology. CIT was quantified in 47% of samples *(n =* 300) up to 4.0 ng/mg Crea (mean = 0.29 ng/mg Crea), whereas DH-CIT was quantified in 21% of samples up to 2.5 ng/mg Crea (mean = 0.39 ng/mg Crea). Considering different age groups, average exposure ranged from 8% to 40% of the provisional tolerable daily intake, whereas four individuals surpassed the limits suggested by the European Food Safety Authority. These results revealed non-negligible exposure levels to CIT, encouraging further investigation in foodstuffs monitoring studies.

## Introduction

Mycotoxins are secondary metabolites produced by several fungi genera, primarily *Aspergillus*, *Penicillium*, *Fusarium*, *Alternaria*, and *Claviceps*. These compounds can be found in cereal grains, food commodities, and animal feed under propitious environmental conditions or because of bad practices at any point from the preharvest interval to the storage.^1,2^ Once ingested, mycotoxins can display a wide variety of adverse effects including immunosuppression, neurotoxicity, or carcinogenicity.^3,4^ In consequence, regulatory authorities set maximum limits (MLs) in certain foodstuffs for several hazardous mycotoxins, in light of tolerable daily intake (TDIs) derived by Scientific Committees, e.g., the European Food Safety Authority or the Joint FAO/WHO Expert Committee on Food Additives.^5^ Over the last years, citrinin (CIT) has become a relevant compound due to its occurrence in grains and grain products and its toxicity,^6,7^ but the EFSA noted that occurrence data are insufficient to conduct dietary exposure assessments for humans.^8^

CIT is produced by several *Aspergillus*, *Penicillium*, and *Monascus* species, and it can be found in stored grain and other plant products like fruits, herbs, and spices, showing a wide distribution throughout different geographical areas around the world and occurring at concentration ranges from a few ng/g up to 1500 ng/g depending on the commodity.^7,9−13^ This toxin has also been identified co-occurring with other toxins produced by these fungi, especially ochratoxin A (OTA).^7,14^ Nonetheless, only the maximum level for citrinin in food supplements based on rice fermented with red yeast *Monascus purpureus* has been set to date.^15^ CIT is a quinone with a planar and conjugated structure that targets primarily the kidney, resulting in necrosis of renal tubules.^8,16^ Although the mechanism responsible for its toxicity is not fully understood, it could be related to the production of reactive oxygen species (ROS) linked to apoptotic processes.^17,18^ Moreover, CIT has genotoxic properties and can induce micronuclei (mainly aneugenic) and chromosomal aberration in several animal and human cell lines.^8,19,20^ The EFSA Contam Panel concluded that the combined effect of OTA and CIT is mainly additive.^8^ In combination with OTA, a synergistic effect has been reported after *in vitro* assays, displaying a higher nephrotoxic^21^ and genotoxicity^22^ potential. Nevertheless, the limited toxicological data available is insufficient to evaluate its carcinogenicity potential, so CIT has been placed into group 3 within the classification released by the International Agency for Research on Cancer (IARC).^23^

Referring to the metabolism of CIT, data are scarce on the sites of its bioconversion and the enzymes involved. The main product of CIT metabolism is dihydrocitrinone (DH-CIT), first detected in rat urine by Dunn et al.^24^ This compound showed a lower cytotoxic and genotoxic potential, so the conversion of CIT to DH-CIT could be considered as a detoxification process.^22^ As regards the bioavailability, little is known in humans. The only toxicokinetic study carried out in humans determined a half-life of 6.7 and 8.9 h for CIT and DH-CIT, respectively, and a rapid absorption of CIT with at least a 40% of the initial dose being excreted in urine.^25^ After metabolization of the parent toxin, the urinary levels of DH-CIT are strongly variable among individuals, with concentrations ranging between 3 and 17 times greater in relation to the parent compound.^26^

According to the toxicological potential of CIT, the EFSA Panel of Food Contaminants derived a provisional tolerable daily intake (PTDI) of 0.2 μg/kg bw per day, which corresponds to the level of no concern for nephrotoxicity in order to characterize the risk of citrinin.^8^ However, considering the lack of data regarding the occurrence of CIT in feed and foodstuffs, a reliable exposure assessment cannot be performed. A complementary approach to assess mycotoxin exposure is biomonitoring, which involves the analysis of parent compounds and/or their metabolites in human biological samples.^27^ In this line, the sum of CIT and DH-CIT in urine has been proposed as an effective biomarker to assess the exposure to CIT.^25,26,28,29^ Several biomonitoring surveys have reported the occurrence of CIT and DH-CIT in urines from different human cohorts from Belgium,^30−32^ Czech Republic,^33^ Portugal,^34,35^ Germany,^36−39^ Haiti,^39^ Bangladesh,^39−43^ Nigeria,^44^ Turkey,^36^ and Tunisia.^45^ Biomarkers should be measured by sensitive and specific analytical methods able to detect even a low level of exposure. Currently, high-resolution mass spectrometry (HRMS) stands as a suitable method for providing accurate measurements at low levels, and its high resolving power ensures a very specific detection in complex mixtures. Hence, the aim of this study was to evaluate the presence of citrinin and dihydrocitrinone in 300 urine samples from the Italian population in order to assess the exposure. For quantification purposes, an ultrahigh-performance liquid chromatography high-resolution mass spectrometry (UHPLC-Q-Orbitrap HRMS)-based methodology was developed.

## Material and Methods

### Chemicals, Reagents, and Materials

Methanol (MeOH), acetonitrile (ACN), and water for the LC mobile phase (LC-MS grade) were purchased from Merck (Darmstadt, Germany). Ammonium formate (analytical grade) was acquired from Fluka (Milan, Italy), and formic acid (MS grade) was provided by Carlo Erba Reagents (Cornaredo, Italy). Sodium chloride (NaCl) and octadecyl carbon chain-bonded silica (C18) (analytical grade) were purchased from Sigma-Aldrich (Milan, Italy). Conical centrifuge polypropylene tubes of 15 mL were obtained from BD Falcon (Milan, Italy). Syringe filters with polytetrafluoroethylene membrane (PTFE, 15 mm, diameter 0.2 μm) were acquired from Phenomenex (Castel Maggiore, Italy).

Analytical standards of CIT and DH-CIT (HPLC purity >98%) were acquired from Sigma-Aldrich (Milan, Italy) and Analyticon Discovery GmbH (Potsdam, Germany), respectively. Stock solutions were prepared diluting 1 mg of each mycotoxin in 1 mL of MeOH. Working solutions were built from the stock, diluting in MeOH/H_2_O (70:30 v/v) 0.1% formic acid until reaching the desired concentrations for spiking experiments. The solutions were stored in tightly closed containers at −20 °C in a well-ventilated place as specified by the manufacturer.

### Urine Sample Collection

First-spot morning urine (50 mL) samples from 300 volunteers living in the Campania region (South Italy) and aged between 2 and 91 years old were collected into sterile plastic vessels during January and February 2018. After collection, each sample was aliquoted and kept at −20 °C until analysis due to stability issues. Volunteers were randomly recruited among students and academic and non-academic staff from the Faculty of Pharmacy of University of Naples Federico II who complied with the following exclusion criteria: (i) only one member per family was allowed; (ii) people exposed to a large number of mycotoxins in a way other than food, such as farmers and veterinarians, were excluded; (iii) people with severe problems in the liver, bile, or kidney could not participate due to related risk of interferences with the metabolism of mycotoxins. The use of medication was not an exclusion criterion since scarce information regarding interferences with mycotoxins is available. The participants were not subjected to any diet restriction before and during the sampling. All volunteers provided a written consent in accordance with the Helsinki Declaration on ethical principles for medical research involving human subjects, and the project was approved by the University of Naples Federico II Institutional Human Research Committee. The sample size (*n =* 300) selected is consistent with previous pilot biomonitoring studies and recommendations from the International Federation of Clinical Chemists (IFCC).^46^

All samples were anonymous, but participants were asked to write down their gender and age in the vessel for sample classification purpose. The sampling tried to maintain the gender parity (male: 45.7%, female: 54.3%). Three age groups were considered: <18 years old (*n =* 20), from 18 to 65 years old *(n =* 170), and >65 years old (*n =* 110). Samples with undetectable levels of mycotoxins were used for recovery studies.

### Sample Preparation

The sample preparation was performed following a previously developed method by Rodríguez-Carrasco et al.^47^ In short, 1.5 mL of the sample was placed into a 2 mL Eppendorf Safe-Lock Microcentrifuge tube and centrifuged at 3926*g* for 3 min. Next, 1 mL of the supernatant was collected and transferred into a 15 mL screw cap test tube with conical bottom and 1 mL of acetonitrile was added. The mixture was vortexed for 30 s, and a mixture of 0.3 g of sodium chloride and 30 mg of C18 sorbent was added. The solution was vortexed for 30 s and centrifuged at 3926*g* for 3 min at 4 °C. Finally, the upper layer was collected and evaporated to dryness under nitrogen flow at 45 °C, reconstituted with 0.5 mL of MeOH/H_2_O (70:30 v/v) 0.1% formic acid and filtered through a 0.2 μm filter prior to UHPLC-Q-Orbitrap HRMS analysis.

### UHPLC-Q-Orbitrap HRMS Analysis

Chromatographic analysis was performed using an ultrahigh-performance liquid chromatograph (UHPLC) Dionex Ultimate 3000 (Thermo Fisher Scientific, Waltham, USA) equipped with a degassing system, an auto sampler device, a quaternary UHPLC pump working at 1250 bar, and a thermostated (30 °C) Luna Omega column (50 × 2.1 mm, 1.6 μm, Phenomenex). The mobile phases were water (A) and methanol (B), both containing 5 mM ammonium formate and 0.1% formic acid. The separation gradient for the UHPLC-Orbitrap HRMS analyses was applied as follows: initial 0% of phase B held for 1 min, increased to 95% in 1 min, and kept for 0.5 min. Next, the gradient switched back to 75% of B in 2.5 min and then decreased again until 60% in 1 min. Finally, the gradient went back to 0% of B in 0.5 min and kept for 1.5 min for column re-equilibration, setting a total run time of 8 min. An aliquot of 5 μL of the sample was injected, and the flow rate was established at 0.4 mL/min.

The UHPLC system was coupled to a Q-Exactive Orbitrap mass spectrometer. The mass spectrometry analysis was simultaneous performed in both positive and negative electrospray (ESI) modes through fast polarity switching, setting two scan events (Full Scan and All Ion Fragmentation, AIF). The ionization parameters were: spray voltage 4 kV (−4 kV in ESI– mode), capillary temperature 290 °C, sheath gas pressure (N_2_ > 95%) 35, auxiliary gas (N_2_ > 95%) 10, auxiliary gas heater temperature 305 °C, S–lens radio frequency (RF) level, 50. Full Scan data collection was performed with the following settings: resolving power 35,000 full-width at half maximum (FWHM) at 200 *m*/*z*, automatic gain control (AGC) target 1 × 10^6^, injection time 200 ms, scan range from 100 to 1000 *m*/*z*, and scan rate 2 scans/s. The parameters for the AIF scan event were as follows: maximum injection time 200 ms, resolving power 17,500 FWHM, AGC target 1 × 10^5^, scan time 0.1 s, scan range from 100 to 1000 *m*/*z*, retention time window, 30 s, and *m*/*z* isolation window 5.0. The UHPLC-Q-Orbitrap parameters were optimized by injection of analytical standards using a solution at 1 μg/mL in both positive and negative ESI modes. A mass tolerance of 5 ppm was set for identification at the intensity threshold of 1000 considering both precursor and product ions. Data analysis was carried out using Quan/Qual Browser Xcalibur v.3.1.66 (Thermo Fisher Scientific, Waltham, USA).

### Method Validation

In-house validation was carried out in accordance with the EU Commission Decision 2002/657/EC.^48^ The assessed parameters were linearity, selectivity, trueness, repeatability, within-laboratory reproducibility, limit of detection (LOD), and limit of quantification (LOQ). Linearity (*r*^2^) was determined through both neat solvent and matrix-matched calibration curves ranging from 25 to 0.01 ng/mL and considering a deviation <20% for each concentration level. In order to evaluate the interference of the matrix, the slopes of both calibration curves were used to calculate the percentage of signal enhancement/suppression (%SSE) through the following equation:
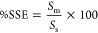
where *S*_m_ represents the matrix-matched calibration slope and *S*_s_ is the solvent calibration slope. An %SSE below 100% indicated signal suppression whereas values above 100% meant signal enhancement in the range of concentrations previously assayed. Trueness was assessed through recovery experiments, spiking blank urine samples at three different concentrations (5, 1, and 0.5 ng/mL). Experiments were performed in triplicate on three non-consecutive days and expressed as intra-day (repeatability, RSD_r_) or inter-day (within-laboratory reproducibility, RSD_R_) relative standard deviation. LODs were established as the lowest concentration where the molecular ion could be distinguished from the background noise (S/N = 3), whereas LOQs were set as the lowest concentration where the molecular ion could be identified inside the linear range, considering a mass error below 5 ppm.

Selectivity was also studied in order to determine the presence of potential coelutants in the matrix, so blanks (*n =* 10) were injected right after the highest calibration sample. For confirmation criteria, the retention times of the analytes in standards and samples were compared.

### Quality Control/Quality Assurance

Chromatographic and spectra data were used for proper confirmation of the analytes. Retention times corresponding to the analytes were compared in both positive samples and standards in neat solvent at a tolerance of ±2.5% of the total run time (8 min). Data quality was monitored using a comprehensive range of quality assurance and quality control procedures. Therefore, a reagent blank, a procedural blank, a replicate sample, and a matrix-matched calibration were included in each batch of samples in order to assess the stability and robustness of the instruments throughout the whole analysis.

### Creatinine Analysis

Urinary levels of creatinine were determined through a spectrophotometric methodology previously reported by Rodríguez-Carrasco et al.^49^ Briefly, 3.5 mM picric acid was mixed with 1000 mM NaOH to obtain alkaline picrate. The resultant solution was kept in dark conditions in an amber glass container. Urine samples were diluted using ultrapure water (1:10, v/v), and 1 mL was reacted with 1 mL of alkaline picrate solution. The optical density was determined after 30 min using a 500 nm Shimadzu mini 1240 spectrophotometer (Shimadzu Corp; Kyoto, Japan). Mycotoxin concentrations were then correlated to the creatinine content of the corresponding sample and expressed as ng/mg Crea.

### Statistical Analysis

For comparison of categorical data, the Pearson chi-square and Fisher exact test were performed in order to assess whether the occurrence of CIT and DH-CIT throughout the different subgroups was significantly different, whereas the Kruskal-Wallis test was used for detecting quantitative differences. A confidence level of 95% was chosen for examining data, and a *p*-value of <0.05 was considered as significant.

## Results and Discussion

### Evaluation of UHPLC-Q-Orbitrap HRMS Conditions

The optimization of the compound-dependent parameters was carried out by injecting analytical standards of CIT and DH-CIT at a concentration of 1 μg/mL. The Q-Orbitrap spectrometer was operated in both positive and negative ESI modes in order to identify the ions with the higher intensity. Table 1 shows the analytical parameters of CIT and DH-CIT referring to elemental composition, retention time, adduct ion, theoretical mass, measured mass, and accuracy. Retention times were 4.78 and 4.97 min for DH-CIT and CIT, respectively. As expected, DH-CIT eluted first, meaning it has a more polar character. Comparing the ionization modes, CIT offered a higher base peak intensity when using positive ESI mode whereas DH-CIT showed a better performance in negative ESI mode. The chosen ions displayed high accuracy when compared to the theoretical masses, with mass errors within the acceptable range (< 5 ppm).

**Table 1:** UHPLC-Q-Orbitrap HRMS Parameters Corresponding to the Analytes

analyte	retention time (min)	elemental composition	adduct ion	theoretical mass (*m*/*z*)	measured mass (*m*/*z*)	accuracy (Δ ppm)
DH-CIT	4.78	C_13_H_14_O_6_	[M – H]^−^	265.07243	265.07241	–0.08
CIT	4.97	C_13_H_14_O_5_	[M + H]^+^	251.09140	251.09129	–0.44

### Method Performance

The proposed method was validated in terms of sensitivity, selectivity, trueness, repeatability (intra-day precision), reproducibility (inter-day precision), linearity, LODs, and LOQs as specified in Commission Decision 2002/657/EC.^48^ Results are shown in Table 2. Both compounds showed correlation coefficients of >0.990 for both neat solvent and matrix-matched calibration curves. A slight signal suppression was calculated, and therefore quantitation based on neat solvent calibration curves was carried out. Recovery results revealed a suitable performance, with values within the acceptable accuracy range of 70–120% at three assayed concentrations, and relative standard deviation <16% for intra-day (RSD_r_) and inter-day (RSD_R_) precision studies were obtained. LODs were established at 0.003 and 0.017 ng/mL for CIT and DH-CIT, respectively, whereas LOQs were set at 0.01 and 0.05 ng/mL for CIT and DH-CIT, respectively. Lastly, the absence of coelutants was confirmed since no peaks were observed in the same retention time zones. Hence, the proposed method was selective, sensitive, and accurate enough for a reliable quantification of CIT and DH-CIT at low ppt levels in urine samples.

**Table 2:** Method Performance Parameters for CIT and DH-CIT^a^

			recovery (%)			precision (%) [RSD_r_, (RSD_R_)]				
analyte	linearity (*r*^2^)	SSE (%)	5 ng/mL	1 ng/mL	0.5 ng/mL	5 ng/mL	1 ng/mL	0.5 ng/mL	LOD (ng/mL)	LOQ (ng/mL)
CIT	0.9987	89	82	86	70	6 (16)	16 (16)	10 (12)	0.003	0.01
DH-CIT	0.9947	94	83	72	72	10 (15)	9 (12)	11 (16)	0.017	0.05

a SSE = signal suppression/enhancement effect; RSD_r_ = intra-day relative standard deviation; RSD_R_ = inter-day relative standard deviation; LOD = limit of detection; LOQ = limit of quantification.

In the literature, there have recently been published analytical methods for the determination of CIT and DH-CIT in human urine, as reviewed in Table 3. The most common extraction procedure is based on immunoaffinity columns, which offer high selectivity for a specific analyte. Nevertheless, considering their cost and the high amount of samples used for a human biomonitoring study, a simpler and more affordable sample preparation that still fits performance parameters is preferred as the salting-out liquid–liquid extraction proposed in the present study. In addition, considering the low concentrations reported in those previous studies, very sensitive analytical methods are required in order to have an accurate overview of CIT and DH-CIT in urine samples. In this line, liquid chromatography coupled to triple quadrupole mass spectrometry has been applied elsewhere for CIT and DH-CIT quantification. High-resolution mass spectrometry methodologies are becoming more usual when analyzing contaminants in complex biological matrices due to its high resolving power and accurate mass measurement.^50,51^ The present study based on a high-resolution mass spectrometry methodology was in-house validated for quantification of CIT and DH-CIT in human urine samples for the first time and applied to determine the occurrence of the studied analytes in 300 human urine samples.

**Table 3:** Human Biomonitoring Studies of CIT Biomarkers in Urine Samples during the Last Decade

			LOQ (ng/mL)		incidence (%)		range (ng/mg Crea)		mean (ng/mg Crea)					
provenance	cohort	no. of samples	CIT	DH-CIT	CIT	DH-CIT	CIT	DH-CIT	CIT	DH-CIT	sample treatment	analytical method	year	reference
Belgium	ana	40	5.76	na	2.5	na	nd-4.5	na	na	na	LLE with SAX SPE clean-up	UHPLC-MS/MS (QQQ)	2012	Njumbe Ediage et al.^30^
Turkey	infants (<2 years)	6	0.05	0.1	100	100	<LOQ-0.20	<LOQ-1.12	na	na	IAC extraction	HPLC-MS/MS (QQQ)	2013	Blaszkewicz et al.^36^
Germany	adults (20–58 years)	4			100	100	<LOQ-0.07	<LOQ-0.34	na	na				
Belgium	adults	32	0.003	0.03	59	66	<LOQ-0.12	<LOQ-0.21	0.026	0.035	IAC extraction	UHPLC-MS/MS (QQQ)	2015	Huybrechts et al.^31^
Belgium	children (3–12 years)	155	0.003	0.03	72	6	<LOQ-0.42	0.27–2.03	0.04	0.81	filter and shoot	UHPLC-MS/MS (QQQ)	2015	Heyndrickx et al.^32^
	adults (19–65 years)	239			59	12	<LOQ-1.49	0.09–2.12	0.07	0.74				
Germany	adults	50	0.05	0.1	82	84	nd-0.19	nd-0.55	0.03	0.1	IAC extraction	HPLC-MS/MS (QQQ)	2015	Ali et al.^37^
Germany	adults	50	na	0.02	na	28	na	<LOQ-0.33	na	0.09	dilute and shoot	HPLC-MS/MS (Q-TRAP)	2015	Gerding et al.^39^
Haiti	adults	142			na	14	na	<LOQ-4.34	na	0.28				
Bangladesh	adults	95			na	75	na	<LOQ-58.82	na	3.12				
Bangladesh	adults (rural area)	32	0.05	0.1	97	91	nd-1.22	nd-7.47	0.14	0.97	IAC extraction	HPLC-MS/MS (QQQ)	2015	Ali et al.^40^
	adults (urban area)	37			92	54	nd-0.45	nd-0.36	0.06	0.08				
Germany	controls (males)	13	0.05	0.1	100	100	<LOQ-0.20	<LOQ-0.57	0.06	0.14	IAC extraction	HPLC-MS/MS (QQQ)	2016	Föllmann et al.^38^
	workers in grain mills (males)	12			100	100	<LOQ-0.06	<LOQ-0.72	0.03	0.19				
	workers in grain mills (females)	5			100	100	<LOQ-0.06	<LOQ-0.38	0.03	0.14				
Bangladesh	pregnant women (rural area)	32	0.05	0.1	84	84	na	na	0.60	0.70	IAC extraction	HPLC-MS/MS (QQQ)	2016	Ali et al.^42^
	pregnant women (suburban area)	22			91	86	na	na	0.39	0.57				
Bangladesh	adults (rural area, summer)	30	0.05	0.1	97	93	nd-1.22	nd-5.39	0.53	2.81	IAC extraction	HPLC-MS/MS (QQQ)	2016	Ali et al.^41^
	adults (rural area, winter)	30			93	97	nd-3.51	nd-46.44	1.10	7.23				
	adults (urban area, summer)	32			90	50	nd-0.45	nd-0.31	0.20	0.31				
	adults (urban area, winter)	32			91	97	nd-5.03	nd-4.64	0.85	2.86				
Portugal	controls	19	na	na	12	2	na	na	na	na	dilute and shoot	HPLC-MS/MS (Q-TRAP)	2018	Viegas et al.^34^
	workers of bread dough company	21			6	3	na	na	na	na				
Nigeria	children (≤8 years), teenagers (9–19 years), adults (≥20 years)	120	na	na	66	58	0.015–241.46	0.05–16.89	5.96	2.39	dilute and shoot	UHPLC-MS/MS (Q-TRAP)	2014	Šarkanj et al.^44^
Czech Republic	kidney tumor patients (40–81 years)	50	0.05	0.1	91	100	<LOQ-0.087	<LOQ-0.160	0.02	0.08	IAC extraction	HPLC-MS/MS (QQQ)	2019	Malir et al.^33^
Portugal	adults	94	1	na	2	na	nd-1.20	na	na	na	QuEChERS-based extraction	UHPLC-MS/MS (QQQ)	2019	Martins et al.^35^
Tunisia	controls	50	0.2	na	72	na	<LOQ-5.72	na	0.53	na	QuEChERS-based extraction	UHPLC-MS/MS (QQQ)	2020	Ouhibi et al.^45^
	colorectal cancer patients	50			76	na	<LOQ-2.94	na	0.95	na				
Bangladesh	infants (<1 years)	49	0.05	0.1	33	82	0.03–0.33	0.06–6.78	1.24	4.59	IAC extraction	HPLC-MS/MS (QQQ)	2020	Ali and Degen^43^
	children (1–6 years)	105			65	98	0.03–3.54	0.06–22.91	1.60	6.15				
Italy	children, teenagers (<18)	20	0.01	0.05	50	25	0.02–1.48	0.41–1.94	0.32	1.04	SALLE	UHPLC-HRMS (Q-Orbitrap)	2021	present study
	adults (18–65)	170			52	25	0.01–4.00	0.05–2.48	0.35	0.37				
	elderly (>65)	110			42	16	0.01–1.39	0.05–1.16	0.19	0.26				
	total	300			47	21	0.01–4.00	0.05–2.48	0.29	0.39				

a Values without creatinine normalization (ng/mL). na = not available; LOQ = limit of quantification; LLE = liquid–liquid extraction; SAX = strong anionic exchange; SPE = solid phase extraction; IAC = immunoaffinity column; SALLE = salting-out assisted liquid–liquid extraction; nd = not detected; HPLC = high-performance liquid chromatography; UHPLC = ultrahigh-performance liquid chromatography; MS/MS = tandem mass spectrometry; HRMS = high-resolution mass spectrometry; Q = quadrupole; QQQ = triple quadrupole.

### Urinary Levels of CIT and DH-CIT in Human Urines

The number of biomarker data for CIT is rather limited compared to its structurally related nephrotoxic mycotoxin ochratoxin A; thus, the detection of CIT in biological samples is of great interest considering the reported synergistic effects when both toxins co-occur.^8,21,22^Table 3 reviews the occurrence data and the concentration of CIT biomarkers in human urines published during the last decade. In this study CIT was detected in 142 out of the 300 analyzed samples (47%) at concentrations ranging from >LOD to 4.00 ng/mg Crea and showing a mean value of 0.29 ng/mg Crea; whereas DH-CIT was detected in 64 out of 300 samples (21%) at levels from >LOD up to 2.48 ng/mg Crea, presenting an average value of 0.39 ng/mg Crea. By age, the excretion ratio CIT:DH-CIT varied from 0.3 (below 18 years old) to 0.9 (between 18 and 65 years), whereas the incidence values of CIT and DH-CIT were comparable throughout the studied age groups. Nonetheless, the DH-CIT average excretion levels (1.04 ng/mg Crea for children, 0.37 ng/mg Crea for adults, and 0.26 ng/mg Crea for elderly) were greater than those CIT levels (0.32, 0.35, and 0.19 ng/mg Crea for children, adults, and elderly, respectively).

**Figure 1:**
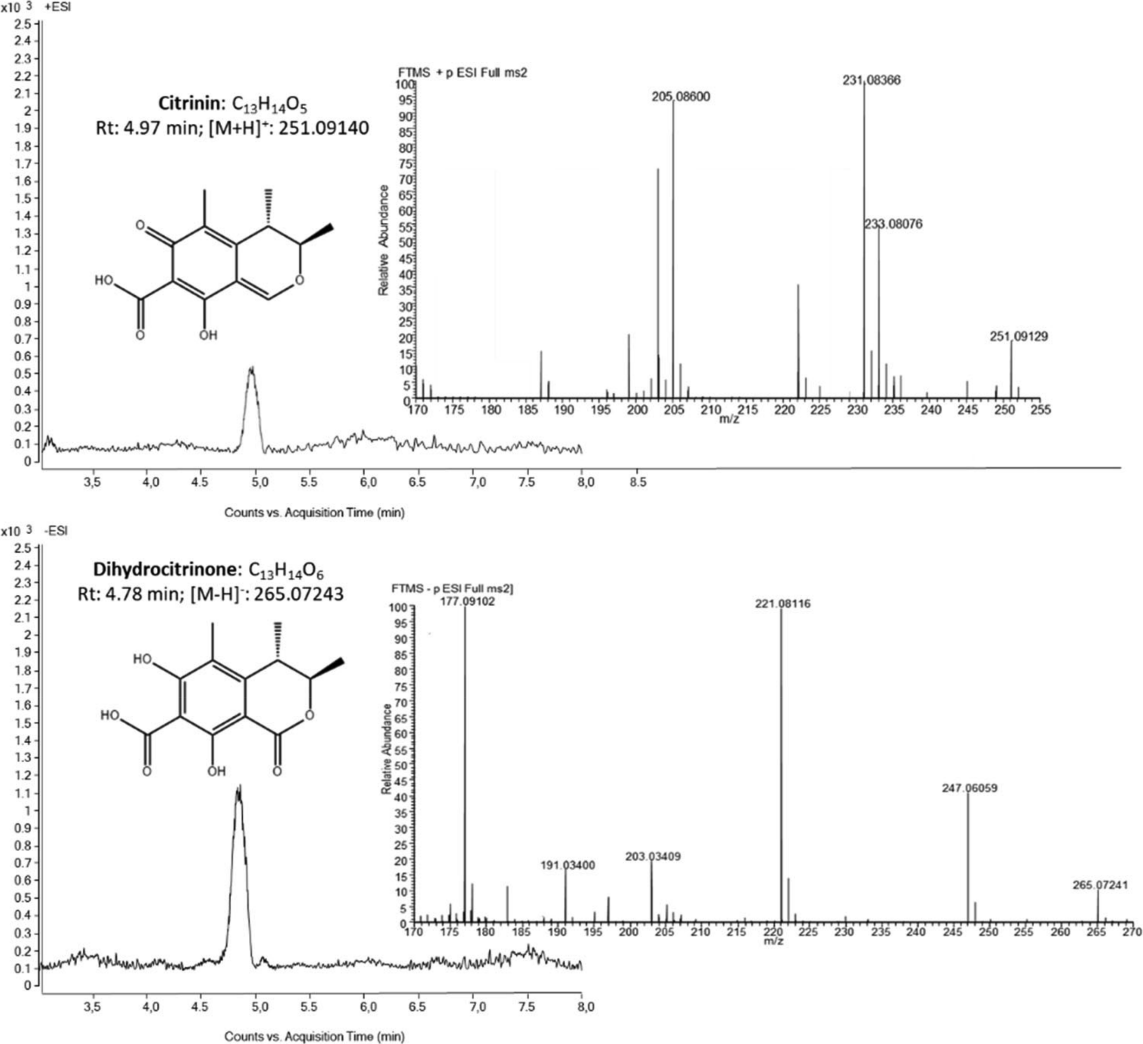
Extracted ion chromatogram and the secondary mass (MS/MS) spectra of a human sample containing citrinin (1.24 ng/mg Crea) and dihydrocitrinone (2.48 ng/mg Crea).

Figure 1 shows the chromatograms and MS/MS spectra extracted from a human urine sample containing citrinin (1.24 ng/mg Crea) and dihydrocitrinone (2.48 ng/mg Crea). In the available literature, the metabolite DH-CIT is often present at higher average levels in urine than the parent compound, although the analyte ratios are quite variable, and this fact justifies the need to measure DH-CIT as an important additional biomarker of CIT exposure.^25,43^ It has to be highlighted that the prevalence of CIT and DH-CIT in here analyzed urine samples was lower than those values reported in the literature for which an incidence of CIT biomarkers >80% were obtained, despite the comparable LOQ levels reported in surveys. However, the here reported CIT and DH-CIT incidences were similar than those reported in biomonitoring studies with a sampling size of over 100.^39,43,44^ Likewise, average values and range of CIT and DH-CIT obtained in these biomonitoring surveys (>100 samples) were comparable with the data obtained in the present work (*n =* 300 samples) (Table 3).

### Estimated Exposure Approach through CIT Biomarkers in Urine

An exposure assessment approach to CIT through urinary data was conducted taken into account the CIT kinetics in humans reported by Degen et al.^25^ who determined that the median value for the excretion of the sum of CIT and DH-CIT was 40.2%. Hence, the following equation was used to calculate the probable daily intake (PDI) of CIT:
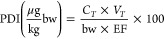
were *C_T_* is the individual urinary total CIT biomarker concentration (ng/mg Crea) obtained in this study; *V_T_* is the average volume of urine excreted in 24 h of 1.6 L;^26^ bw is the body weight provided in the inform consent from each participant, and EF is the daily urinary CIT excretion rate of 40.2% (the median fraction of an oral CIT dose excreted within 24 h).^25^

Based on the above, the CIT PDI for each participant was calculated and PDI values were compared with the provisional tolerable daily intake (PTDI) of 0.2 μg/kg bw, the level of no concern for nephrotoxicity set by the EFSA.^8^ Results are shown in Table 4. Matching the calculated data with the CIT PTDI value, it comes out that the resulting Italian average exposures represents a range between 8% and 40%, being children the most exposed population group. However, 6.4% of the positive tested subjects (*n =* 142) had biomarkers levels, which were indicative of a CIT exposure comparable to half the value considered as PTDI, and four individuals surpassed the limits suggested by the EFSA.

**Table 4:** Exposure Assessment Approach to CIT Based on CIT Urinary Biomarkers

	CIT biomarkers (sum of CIT and DH-CIT, ng/mg Crea)			probable daily intakes (PDI, μg/kg bw)			Percentage of provisional tolerable daily intakes (PTDI)		
population group	CIT_min_	aCIT_mean_	CIT_max_	PDI_min_	PDI_mean_	PDI_max_	PTDI_min_	PTDI_mean_	PTDI_max_
children (<18 years)	0.003	0.842	3.412	0.069	0.095	0.242	0.2	40	113
adults (≥18 and ≤65 years)	0.008	0.526	4.723	0.007	0.030	0.268	0.2	15	134
elderly (>65 years)	0.003	0.293	1.391	0.011	0.017	0.071	0.1	8	40

a Average value based on positive samples only. CIT_min_ and CIT_max_ indicate the lowest and highest concentration of CIT total found in urines according to each population group; CIT_mean_ indicates the mean CIT total values for each population group; PDI_min_ and PDI_max_ indicate the range of CIT PDIs; PDI_mean_ indicates the CIT PDI based on mean CIT total levels found in urine; PTDI_min_ and PTDI_max_ indicate the range of exposure to CIT for each population group; PTDI_mean_ indicate exposure to CIT based on mean CIT total levels found in urine.

As individual data from other surveys were not available to us, it was not possible to calculate their CIT PDIs. Nonetheless, average CIT and DH-CIT urinary levels reported by some European studies were used to estimate exposure to CIT through biomarkers data (Table 3). Heyndrickx et al.^32^ conducted a Belgian biomonitoring study, and they reported average levels of CIT (0.040 ng/mg Crea and 0.074 ng/mg Crea) and DH-CIT (0.810 ng/mg Crea and 0.739 ng/mg Crea) for children (*n =* 155) and adults (*n =* 239), respectively. Those data were used to calculate CIT total as an approach to obtain CIT PDI for both population groups. In this approach, body weight values of 70 and 21.7 kg were assumed for adults (>18 years) and children (3–10 years) as suggested by the EFSA.^8^ Based on these assumptions, calculated exposures were 23% CIT PTDI for adults and 53% CIT PTDI for children. Similarly, exposure estimates for German adults (*n =* 50) were calculated according to CIT (0.034 ng/mg Crea) and DH-CIT (0.102 ng/mg Crea) urinary data reported by Ali et al.,^37^ corresponding to an exposure to CIT equivalent to 23% PTDI. Hence, the obtained results showed a widespread human exposure to CIT. Non-negligible exposure levels to CIT were highlighted in this study and data was comparable to other European biomonitoring studies. In addition, the safe levels (PTDI) surpassed by some individuals could raise concern and should trigger more efforts to analyze and monitor the CIT levels in foodstuffs in future studies. Results here reported are based on a CIT excretion rate assumption and thus, inter-individual variations, derived from different metabolism activities, should be taken into account. Moreover, the excretion rate may also vary in the same subject.

To conclude, a biomonitoring study on CIT and its metabolite DH-CIT in 300 urine samples from the Italian population was carried out through an ultrahigh-performance liquid chromatography coupled to high-resolution Q-Orbitrap mass spectrometry methodology for the first time. Samples were extracted using a salting-out assisted liquid–liquid extraction alongside a simple clean-up step based on C18. This procedure was validated according to EU Commission Decision 2002/657/EC in terms linearity, selectivity, trueness, repeatability, within-laboratory reproducibility, LOD, and LOQ. CIT was detected in 47% of the samples (*n =* 300) at concentrations ranging from >LOD to 4.0 ng/mg Crea (mean value = 0.29 ng/mg Crea), whereas DH-CIT was detected in 21% of samples (*n =* 300) at levels from >LOD up to 2.5 ng/mg Crea (mean value = 0.39 ng/mg Crea). These results are comparable with previous biomonitoring studies including a large sampling (*n* > 100). The exposure of the Italian population to CIT was estimated using the sum of CIT and DH-CIT as a biomarker. Considering the different age groups, CIT average exposure ranged from 8% to 40% of the PTDI, being children the most exposed group, whereas four individuals surpassed the limits suggested by the EFSA. A similar approach was used for estimating the exposure using data from previous European biomonitoring studies, showing similar PTDI values. Hence, these results revealed non-negligible exposure levels to CIT within the Italian population and comparable to previous European studies. The surpassing of the safety levels could raise concern, encouraging further CIT investigation in foodstuffs monitoring studies.

## Abbreviations Used

UHPLC: ultrahigh-performance liquid chromatography
HRMS: high-resolution mass spectrometry
PTDI: provisional tolerable daily intake
ML: maximum limit
RSD_R_: inter-day relative standard deviation
RSD_r_: intra-day relative standard deviation
TDI: tolerable daily intake
CIT: citrinin
DH-CIT: dihydrocitrinone
ROS: reactive oxygen species
OTA: ochratoxin A
PDI: probable daily intake
bw: body weight
EF: urinary CIT excretion ratio
MeOH: methanol
ACN: acetonitrile
C18: octadecyl carbon chain-bonded silica
ESI: electrospray ionization
AIF: all ion fragmentation
FWHM: full-width at half maximum
AGC: automatic gain control
LOQ: limit of quantification
LOD: limit of detection
SSE: signal suppression/enhancement
S/N: signal-to-noise ratio
